# Excessive Nitrogen Fertilization Favors the Colonization, Survival, and Development of *Sogatella furcifera* via Bottom-Up Effects

**DOI:** 10.3390/plants10050875

**Published:** 2021-04-27

**Authors:** Zaiyuan Li, Bo Xu, Tianhua Du, Yuekun Ma, Xiaohai Tian, Fulian Wang, Wenkai Wang

**Affiliations:** Forewarning and Management of Agricultural and Forestry Pests, Hubei Engineering Technology Center, Institute of Entomological Science, College of Agriculture, Yangtze University, Jingzhou 434025, China; zaiyuanli01@163.com (Z.L.); 15327394823@163.com (B.X.); dutianhua01@163.com (T.D.); harris_m@163.com (Y.M.); xiaohait@sina.com (X.T.)

**Keywords:** rice-planthopper, nitrogen levels, bottom-up effects, nutritional interaction, IPM

## Abstract

Fertilization can trigger bottom-up effects on crop plant–insect pest interactions. The intensive use of nitrogen fertilizer has been a common practice in rice production, while the yield has long been challenged by the white-backed planthopper, *Sogatella furcifera* (Horváth). High nitrogen fertilization can facilitate *S. furcifera* infestation, however, how nitrogen fertilizer leads to high *S. furcifera* infestation and the nutritional interactions between rice and *S. furcifera* are poorly understood. Here, we evaluated the effects of various levels of nitrogen fertilizer application (0–350 kg/ha) on rice, and subsequently on *S. furcifera* performance. We found that higher nitrogen fertilizer application: (1) increases the preference of infestation behaviors (feeding and oviposition), (2) extends infestation time (adult lifespan), and (3) shortens generation reproduction time (nymph, pre-oviposition, and egg period), which explain the high *S. furcifera* infestation ratio on rice paddies under high nitrogen conditions. Moreover, high nitrogen fertilizer application increased all tested rice physical indexes (plant height, leaf area, and leaf width) and physiological indexes (chlorophyll content, water content, dry matter mass, and soluble protein content), except for leaf thickness, which was reduced. Correlation analysis indicated that the specific rice physical and/or physiological indexes were conducive to the increased infestation behavior preference, extended infestation time, and shortened generation reproduction time of *S. furcifera*. The results suggested that nitrogen fertilizer triggers bottom-up effects on rice and increases *S. furcifera* populations. The present study provides an insight into how excess nitrogen fertilization shapes rice–planthopper interactions and the consequent positive effect on *S. furcifera* infestation.

## 1. Introduction

Plant–arthropod interactions are thought of as an important question in ecological research, which can provide insight into the dynamics of ecological communities and the mechanisms that shape interactions in complex food webs [1,2]. Plant–arthropod interactions can be markedly shaped by bottom-up forces, which are affected by abiotic factors and can in turn influence the performance of insect herbivores [1,3,4,5,6,7]. Plant nutrients could impact the performance of herbivorous insects via changes in plant quality in terms of nutritional and defensive aspects and determine bottom-up effects on plant–insect herbivore interactions. Among the plant nutritional factors that influence the performance of insect herbivores to a crop is total nitrogen [8,9,10].

Due to a large difference in nitrogen content between herbivorous insects and plant tissues, the nitrogen-containing nutrients of host plants are frequently considered as a limiting resource for the population development of herbivore insects [11]. The nitrogen content of plants is often regulated by nitrogen fertilizer application [11]. Insufficient nitrogen input to plants was shown to impair the performance of herbivore insects, which was termed the “nitrogen limitation hypothesis” [5,12]. The harmful effects on the performance of herbivorous insects may be caused by the accumulation of high plant allelochemicals and toxic substances found on nitrogen-deficient plants [4,5,10]. Nitrogen-rich plants have positive effects on herbivore insects’ performance, probably owing to deposition-induced improvements in host plant chemistry, which can significantly affect the structure and nutrition of plants [13,14,15]. Most current studies have shown support for the ‘‘nitrogen limitation hypothesis” [16,17,18]. However, several studies show that the development and survival of herbivore insects could respond negatively to nitrogen inputs to host plants, which undermined the generality of the “nitrogen limitation hypothesis” [19,20,21,22].

Rice (*Oryza sativa* L.) is one of the top five carbohydrate crops for the world’s human population, especially in Asia. It is a major staple food, which supports more than three billion people and represents 50% to 80% of their daily calorie intake [23]. Part of the progress in rice production has resulted from a large amount of chemical fertilizer input, especially nitrogen (N) [24]. Low soil nitrogen level, especially in depleted croplands, is considered a major limitation for the growth of rice [16]. Therefore, the intensive use of nitrogen fertilizer has been a common approach for pursuing higher crop yields, and nitrogen application in rice production has shown a gradually increasing trend, as high as 350 kg/ha in many Asian regions, exceeding the reasonable amounts of 150 to 250 kg/ha [25,26]. However, rice is attacked by about 800 species of insect pests in the field and postharvest storage [27]. The white-backed planthopper, *Sogatella furcifera* (Horváth) (Hemiptera: Delphacidae), one of the most destructive migratory pests of rice, poses a substantial threat to rice production. Notably, Zhou et al. [28] found that the infestation ratio of *S. furcifera* was higher in paddies with high nitrogen fertilizer application. However, how high nitrogen fertilization level leads to high *S. furcifera* infestation and the nutritional interactions between rice and *S. furcifera* are poorly understood.

In the present study, we hypothesize that rice subjected to varying nitrogen inputs may trigger bottom-up effects on behavior as well as population fitness of *S. furcifera*. To test our hypothesis, we set up a trophic “rice-planthopper” system to carry out a series of bioassays under laboratory conditions, including (1) feeding and oviposition preferences of *S. furcifera*, (2) the life parameters of *S. furcifera*, (3) the physical and physiological indexes of rice, and (4) the relationships between behavior preferences, life parameters of *S. furcifera,* and physical and physiological indexes of rice with different nitrogen fertilizer applications. With these, we provide an insight into how excess nitrogen fertilization shapes rice–planthopper nutritional interactions and the consequent potential positive effect on *S. furcifera* infestation. Furthermore, these results may help to optimize the integrated pest management (IPM) of *S. furcifera* by nitrogen fertilizer manipulation.

## 2. Results

### 2.1. Feeding and Oviposition Preferences of S. furcifera Adults

**Figure 1:**
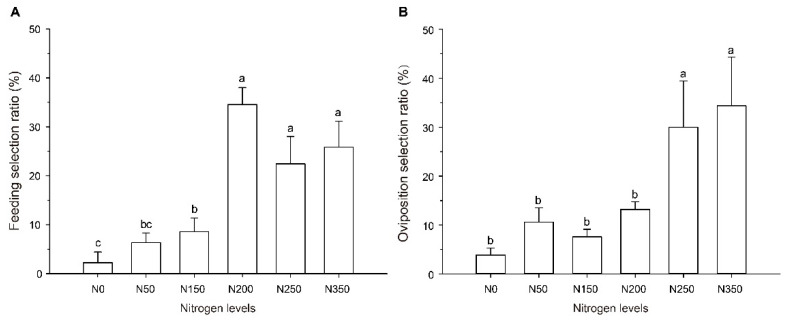
Feeding and oviposition preference of *S. furcifera* on rice of different nitrogen levels. (**A**) Feeding preference of *S. furcifera* on rice of different nitrogen levels. Feeding selection ratio of each nitrogen fertility level is expressed as a percent of the adults settling on a specific fertilizer level plant. (**B**) Oviposition preference of female on rice of different nitrogen levels. Oviposition selection ratio of each nitrogen fertility level is expressed as a percent of the eggs laid on a specific fertilizer level plant. In panels **A** and **B**, values (means ± SE) labeled with different letters are significantly different at *p* < 0.05 (one-way ANOVA, LSD multiple range test, *p* < 0.05). N0, N50, N150, N200, N250, and N350 represent the rice plants grown from the six nitrogen fertility levels (0, 50, 150, 200, 250, and 350 kg/ha), respectively.

The high amount of nitrogen fertilizer applied to rice plants had a significant effect on the feeding behavior of *S. furcifera* (*F* = 11.546, *df* = 5, 29, *p* < 0.001; Figure 1A). The adults of *S. furcifera* preferred to feed on N200, N250, and N350 rice plants, compared with N0 rice plants, and the adults’ settling selection ratio increased by 32.33%, 20.22%, and 23.67% on rice from N200, N250, and N350, respectively.

The high nitrogen fertilizer levels also influenced the oviposition behavior of the adult females (*F* = 7.302, *df* = 5, 17, *p* = 0.002; Figure 1B). Among all the rice plants provided as options, the females preferred to lay eggs on the N250 and N350 rice plants, laying 677.78% and 791.36% respectively, more eggs than on N0 rice plants.

### 2.2. Life Parameters of S. furcifera on Rice with Different Nitrogen Fertilizer Levels

#### 2.2.1. Nymph Period

The nymph period differed in rice with different nitrogen fertilizer applications (*F* = 24.837, *df* = 5, 245, *p* < 0.001; Table 1) and the nymph period was shortened with nitrogen fertilizer application. The nymph period of *S. furcifera* raised on the N0 rice was the longest, being significantly longer (*p* < 0.001) than nymphs fed on the N50, N150, N200, N250, and N350 rice. The shorter nymph periods were observed on the nymphs raised on the high-nitrogen (N250 and N350) rice plants.

#### 2.2.2. Longevity of Adults

There was a significant increase in the adult longevity of *S. furcifera* with high nitrogen fertilizer application (*F* = 4.815, *df* = 5, 243, *p* < 0.001; Table 1). Adult longevity of the insects raised on N0 rice was the shortest, which was significantly lower than the longevity of the adults raised on the N150 (*p* < 0.001), N200 (*p* < 0.001), N250 (*p* < 0.001), and N350 (*p* = 0.011) rice plants.

#### 2.2.3. Mortality Dynamic of Adults

**Figure 2:**
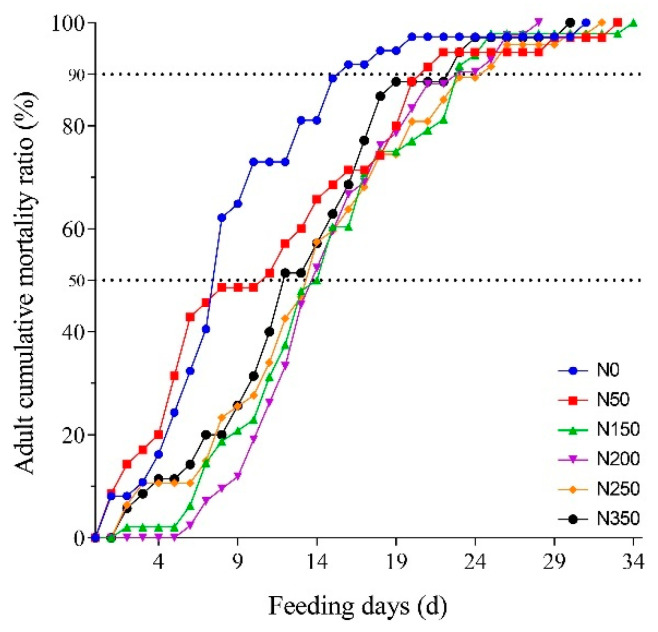
Adult cumulative mortality of *S. furcifera* on rice with different nitrogen fertilizer applications.

Adult mortality varied in *S. furcifera* insects raised on rice with different nitrogen fertilizer application levels (Figure 2). The relationship between the length of feeding and adult cumulative mortality ratio was determined by a logistic relationship and was described by the nonlinear regression (Table 2 and Table 3). The mortality ratio of the adult was highest in the insects reared on the N0 and N50 rice plants (Figure 2). When causing 50% adult cumulative mortality, the feeding day (7.9 days) was the shortest on the N0, whereas the feeding days were longer when adults were fed on the rice of N50, N150, N200, N250, and N350 (Table 4). Ingestion of high-nitrogen rice extended the lifespan of the adult stage. In addition, the time causing 50% adult cumulative mortality was delayed by 6.2, 6.2, 5.7, and 4.7 days when adults fed on rice of N150, N200, N250, and N350 compared with that on N0, respectively. The time causing 90% adult cumulative mortality was observed to be the shortest on N0 rice plants (Table 4).

#### 2.2.4. Pre-Oviposition Period of Female

The pre-oviposition period lasted the longest in the females fed on the N0 rice plant, compared with those fed on the N200 (*p* = 0.007) and N250 (*p* = 0.044) (Table 1).

#### 2.2.5. Egg Period

The developmental duration of the eggs of *S. furcifera* was established. The developmental period (*F* = 45.891, *df* = 5, 882, *p* = 0.000; Table 1) was significantly shortened by nitrogen fertilizer application. The longest developmental period was recorded for eggs from females fed N0 rice plants. Developmental durations of eggs were significantly shorter for eggs laid by females fed with the N150 (*p* = 0.002), N200 (*p* < 0.001), N250 (*p* < 0.001), and N350 (*p* < 0.001).

#### 2.2.6. Biological Cycle Duration

The development duration from egg to first oviposition varied significantly with different nitrogen fertilizer applications (*F* = 22.456, *df* = 5, 23, *p* < 0.001; Table 1). The average total pre-oviposition period varied from 28.27 days on N0 to 23.27 days on N250. The total pre-oviposition period of *S. furcifera* fed with nitrogen application (N50–N350) rice plants was significantly shorter than N0 rice (*P*_N50-vs-N0_ = 0.002; *P*_N150-vs-N0_ < 0.001; *P*_N200-vs-N0_ < 0.001; *P*_N250-vs-N0_ < 0.001; *P*_N350-vs-N0_ < 0.001). However, the total pre-oviposition periods had no significant difference when *S. furcifera* was fed with N150, N200, N250, and N350 rice plants (*p* > 0.114).

### 2.3. Rice Physical Parameters with Different Nitrogen Fertilizer Applications

#### 2.3.1. Plant Height

The effects of nitrogen application on plant height are listed in Table 5. The heights of the rice plant were significantly affected by the nitrogen application levels (*F* = 8.577, *df* = 5, 59, *p* < 0.001). The lowest plant height was measured in the N0 rice, which was significantly lower than that of the rice plants of N150 (*p* < 0.001), N200 (*p* < 0.001), N250 (*p* < 0.001), and N350 (*p* < 0.001).

#### 2.3.2. Leaf Area, Width, and Thickness

The rice leaf area significantly varied with the levels of nitrogen fertilizer application (*F* = 9.864, *df* = 5, 23, *p* < 0.001; Table 5). The smallest leaf area measured was 2.0 cm^2^ in the N0 rice plant, which had significantly smaller leaf areas than that of N150, N200, N250, and N350 plants (*p* < 0.036). The largest leaf areas measured were 2.9 and 3.1 cm^2^ in the N250 and N350 rice plants, respectively.

Similarly, the differences in leaf width were evident when the rice was provided with different amounts of nitrogen fertilizer (*F* = 9.864, *df* = 5, 23, *p* < 0.001; Table 5). The leaf width of rice significantly increased with the increase of nitrogen fertilizer application.

Nitrogen fertilizer application greatly reduced the leaf thickness of rice (*F* = 5.291, *df* = 5, 23, *p* = 0.004; Table 5). The leaf thickness of the N0 rice was measured as 12.20 mg/cm^2^, which was significantly thicker than the rice plants amended with nitrogen fertilizer (*p* < 0.044).

### 2.4. Rice Physiological Parameters with Different Nitrogen Fertilizer Applications

#### 2.4.1. Leaf Chlorophyll Content (SPAD)

There was a significant increase in the leaf chlorophyll content (SPAD) with increased levels of nitrogen fertilizer amendment (*F* = 37.482, *df* = 5, 59, *p* < 0.001; Table 6). It was highest in the N250 and N350 treatments and significantly dropped in the N0 treatment (*p* < 0.001).

#### 2.4.2. Water Content, Dry Matter Mass, and Soluble Protein Content

Water content in the main stems of the rice plant increased significantly (*F* = 10.240, *df* = 5, 29, *p* < 0.001; Table 6) with the increased amount of nitrogen fertilizer available to rice plants. The water content of the N0 rice was significantly lower than that of other rice plants given nitrogen fertilizer (*p* < 0.037). The water content in N200, N250, and N350 was significantly higher than N50 (*p* < 0.026).

Dry matter mass also differed significantly (*F* = 21.501, *df* = 5, 29, *p* < 0.001; Table 6) among the rice plants receiving various nitrogen fertilizer treatments, and nitrogen fertilizer application increased the rice ground dry matter, with the highest in the N200 and N350 and the lowest in N0 rice plants.

Rice leaf soluble protein contents significantly differed across the six nitrogen fertilizer treatments (*F* = 11.849, *df* = 5, 23, *p* = 0.000; Table 6). It was higher in the N350 and N250 rice leaves than in the others, and it steadily declined in the leaves of rice plants grown in the soil with no or less nitrogen fertilizer incorporated.

### 2.5. Relationships between Feeding and Oviposition Preferences, Life Parameters of S. furcifera, and Parameters of Rice with Different Nitrogen Fertilizer Applications

The relationships between feeding and oviposition preferences, life parameters of *S. furcifera,* and the plant parameters of rice with different nitrogen fertilizer levels were determined with the Pearson correlation analysis (Table 7). The results indicated a significant positive correlation between the feeding preference and chlorophyll content (SPAD). Oviposition preference had significant positive correlations with leaf area, leaf width, and soluble protein content. The nymph period had significant negative correlations with the plant height, leaf area, leaf width, leaf thickness, chlorophyll content (SPAD), water content, dry matter mass, and soluble protein content. Adult longevity had significant positive correlations with plant height, chlorophyll content (SPAD), water content, and dry matter mass; however, it had a negative correlation with leaf thickness. The female pre-oviposition period indicated only a significant negative correlation with plant height and water content. There were significant negative correlations between the egg period with chlorophyll content (SPAD), water content, and soluble protein content.

## 3. Discussion

In the present study, our results demonstrated that variation of nitrogen input to rice plants significantly affected the behaviors and life performance of *S. furcifera*. We found that: (1) higher nitrogen fertilizer application to rice plants increased the feeding and oviposition preferences of adults, and (2) nitrogen fertilizer application extended the adults’ longevity and shortened the generation reproduction time of *S. furcifera*. These results indicated that altered nitrogen inputs to rice plants trigger a bottom-up effect on the biological traits of *S. furcifera* and then may explain the high infestation ratio of rice paddies with high nitrogen fertilizer by *S. furcifera*. The physical and physiological indexes change in host plants further explained the effects on the biological traits of *S. furcifera*. Nitrogen fertilizer application increased physical indexes (including plant height, leaf area, and leaf width) and physiological indexes (including chlorophyll content, water content, dry matter mass, and soluble protein content) in rice.

To understand insect behaviors, it is necessary to predict and prevent insect infestation outbreaks [29]. Feeding behavior is the first stage of acquiring nutrients for herbivorous insects, which is the foundation for a series of physiological activities to maintain the insect population. Oviposition behavior, the last stage of an herbivore’s life cycle, has a crucial influence on the nutrition of the next-generation larvae [30,31,32]. Host orientation is a process by which organisms select host plants with potential nutritional resources, and it plays an important role in the process of feeding and oviposition [1]. In this study, nitrogen input to rice increased the feeding and oviposition preference of *S. furcifera* adults (Figure 1), especially in high nitrogen (N250, 350). In other words, excessive nitrogen fertilization favors the colonization of *S. furcifera*, which in turn leads to a high infection rate in the paddy. We assumed that the rice plant quality was improved under nitrogen input, thus causing the behavior preference. Indeed, we found that the physical and physiological parameters of rice were improved with nitrogen fertilizer applications (Table 5 and Table 6). In particular, there was a significant correlation between the rice chlorophyll content (SPAD) and feeding preference after nitrogen application (Table 7). The chlorophyll content was positively correlated with nitrogen content and could be regarded as an indicator of the nitrogen content of plants [33]. Therefore, the nitrogen content of rice plants plays an important role in the feeding orientation of *S. furcifera* adults. Similarly, the leaf area, leaf width, and soluble protein content may play more important roles in oviposition orientation. In addition, most insects rely on olfactory cues to determine their host orientation [34]. We assumed that volatile organic compounds (VOCs) changed under nitrogen input, thus causing the behavior preference of *S. furcifera*. For example, tomato plants released less volatiles and increased the preference of *Bemisia tabaci* after high-nitrogen treatment [35].

For herbivorous insects, nitrogen plays a role in plant–herbivore interactions and the insects’ development. Nitrogen fertilizer application on host plants not only influences many aspects of the behavior of herbivorous insects [36], but also impacts consumer survival, development rates, and fecundity by bottom-up effects after colonization [37,38]. The complete development of herbivorous insects depends on whether they obtain sufficient nutrients from the host plants [39,40]. In our study, nitrogen inputs positively affect *S. furcifera* survival and development (Table 1). In other words, excessive nitrogen fertilization favors the survival and development of *S. furcifera*. Furthermore, the rice nutrients (including chlorophyll content, water content, dry matter mass, and soluble protein content) were significantly increased in high nitrogen. Organic nitrogen (including amino acids and proteins) is considered as a limiting nutrient factor for herbivorous insects. When herbivorous insects feed on nitrogen-deficient plants, their metabolism can be reduced or impaired during the critical growth period [5,8]. Here, the developmental duration of *S. furcifera* egg and nymph had significant negative correlations with rice chlorophyll content (SPAD) and soluble protein content (Table 7). Therefore, we suggest that chlorophyll content (SPAD) and soluble protein content were key regulated factors influencing *S. furcifera*. Besides, it is worth noting that under nitrogen fertilizer, the water content has a significant correlation with all life parameters of *S. furcifera*. Similarly, Han et al. [5] reported that *Tuta absoluta* had a lower survival rate and longer development time when fed on the host plants subjected to drought. We assumed that *S. furcifera* had difficulty in obtaining enough water for optimal development, thus causing a slow development when fed on the nitrogen-deficient rice plants. In addition, we found that the development time of *S. furcifera* from egg to first oviposition did not significantly shorten when fed on N150–N350 rice plants. Similar results were found in *Aphis gossypii* [41] and *Trialeurodes vaporariorum* [42]. The total pre-oviposition period of *T. vaporariorum* was not significantly different fed on a nitrogen concentration of more than 310 ppm in the nutrient solution of the tomato plants [42]. We assumed that the *S. furcifera* may excrete excess nutrients from its body when they fed on N150–N350 rice plants [43].

Mechanical barriers can reduce herbivores’ ingestion performance, as they increase plant resistance [44]. Zheng et al. [45] reported that *Laodelphax striatellus* preferred to feed on rice with a large leaf area. In this study, we found that the plant height, leaf area, and leaf blade width of rice significantly increased after nitrogen fertilizer application, which may help the *S. furcifera* to select its optimal host. Besides, leaf thickness is related to a decline in the ingestion ability of sap-sucking insects [46,47,48]. For example, *Aphis gossypii* needed to spend more time penetrating thick cotton leaves, which resulted in reduction of feeding efficiency [49]. Similarly, the penetration of mature lemon leaves by *Parabemisia myricae* nymphs appeared to be inhibited by cuticle thickness [50]. In this study, we found that there was a negative correlation between the leaf thickness and the levels of nitrogen fertilizer application in rice. We suggest that the leaf thickness of rice decreases after nitrogen fertilizer application, and it reduces the mechanical barrier on ingestion by *S. furcifera*. Therefore, *S. furcifera* can get more nutrition from high-nitrogen plants, which subsequently favors the survival and development of *S. furcifera*.

Adult life expectancies significantly increased when raised on rice with nitrogen fertilizer application, showing a positive correlation between adult lifespan and nitrogen fertilizer application. The times causing 50% and 90% adult cumulative mortality were extended for *S. furcifera* in the rice with high nitrogen fertilizer application (Table 4). Huang and Feng [51] found that *S. furcifera* adults consume more than nymphs, indicating that the adult stage was the most harmful period for rice. Our results suggest that nitrogen input may extend the adult lifespan and thus cause a longer rice infestation time, which may be one of the reasons why the rice with high-nitrogen fertilizer application experienced more damage than the one with low application [52]. In addition, the rice with high-nitrogen fertilizer application shortened the length of the nymph, pre-oviposition, and egg stages, thus shortening the life cycle of *S. furcifera*, however, increasing fecundity and total populations. Therefore, this may be another reason for the high infestation rate of *S. furcifera* populations in the paddy with high-nitrogen fertilizer.

To conclude, we suggest that nitrogen input to rice improves plant quality (physiological and physical indexes) and further facilitates the colonization, survival, and development of *S. furcifera*. Our findings support the “plant vigor hypothesis”, that vigorous plants are the best and preferred host plants for herbivore insects [5,53].

Insufficient nitrogen input to plants triggered negative bottom-up effects on *S. furcifera*, which was consistent with the “nitrogen limitation hypothesis” [54,55]. Manipulating nitrogen fertilization inputs could help to optimize agronomic practices against herbivore pests [7]. This method could gain optimal ecological and economic benefits based on a little damage to plants and negligible crop yield losses. Based on a complex agronomic database covering a wide range of climate conditions, soil types, and field managements, Che et al. [56] found that in most rice-growing areas, rice grain yield with nitrogen inputs of 200–250 kg/ha was higher than other nitrogen rate inputs. Here, the preference of infestation behaviors (feeding and oviposition), the infestation time (adult lifespan), and generation reproduction time (nymph, pre-oviposition, and egg period) of *S. furcifera* in 200 kg/ha nitrogen input were sub-optimal compared to high-nitrogen inputs (250 and 350 kg/ha). Therefore, we propose 200 kg/ha nitrogen input as an optimal fertilizer level in rice field managements since this will create a win-win situation. Moreover, rice cultivars that are tolerant to nitrogen-deficiency stress were deliberately selected in breeding, underpinning agronomic leverage for IPM packages against *S. furcifera* by the bottom-up effect.

## 4. Materials and Methods

### 4.1. Preparation of Soil with Various Fertility Levels

Soil for planting the rice plants was obtained from the fields at the Yangtze University Experimental Station for Crop Pests (30°35′50″ N, 112°14′77″ E) in Hubei, China. To prepare the planting soil with specific fertility levels, the dry soil was mixed with urea fertilizer (soluble nitrogen ≥ 46.4%, Hubei Sanning Chemical Co., Ltd., Zhijiang, China) at the rate of 0, 50, 150, 200, 250, and 350 kg/ha. This converted to 0, 0.033, 0.098, 0.131, 0.164, or 0.229 g urea respectively, mixed with 456.50 cm^3^ dry soil to fill each pot (diameter 7 cm, height 14 cm). Thirty pots for each of the above six fertility levels were prepared for planting rice, and a batch of 180 pots was prepared every 10 days to ensure enough pots were available for planting rice plants for the experiment.

### 4.2. Rice Plants’ Preparation for Nitrogen Treatments

Germinated rice seeds of the variety Taichung Native 1 (TN1), susceptible to *S. furcifera*, were individually planted (one seed per pot) in each prepared pot already filled with soil amended with fertilizer. Thirty single-seed pots were prepared and repeated every ten days, for each fertility level, to ensure enough rice plants for the experiment, and they were then kept in cages, measuring 60 × 80 × 60 cm^3^, under laboratory conditions (26 ± 1 °C, 70–80% RH, and 14 h light). Seeded pots were watered as needed and the water level was kept below the upper edge of each pot. Pesticides were not used on the rice plants throughout the experiment. The rice plants grown from the six fertility levels were called N0, N50, N150, N200, N250, and N350 rice, respectively. The rice plants were used in the experiments, 35 days after transplanting (DAT).

### 4.3. Insects

A colony of *S. furcifera* was kindly provided by Dr. Hou of the Chinese Academy of Agricultural Sciences. To maintain and increase the test insect population, they were reared on the N0 rice plants without exposure to any insecticide. The rice plants were enclosed in 80-mesh screen cages (30 × 30 × 60 cm^3^) placed in a climate-controlled rearing room with 26 ± 1 °C, 70 ± 5% RH, and a photoperiod of 14L:10D.

Under the same conditions mentioned above, *S. furcifera* was also reared on N50, N150, N200, N250, and N350 rice plants in order to obtain test insects that were reared at various nitrogen fertility levels. Newly hatched nymphs (<24 h) and newly emerged adults (<24 h) were used in the experiments, except where otherwise specifically noted.

### 4.4. Feeding and Oviposition Preferences

To prepare for the experiment testing rice nitrogen levels on the feeding and oviposition preference of *S. furcifera*, one 35 DAT potted rice plant from each of the six nitrogen levels (N0, N50, N150 N200, N250, and N350) was randomly selected to be included in the test. The selected rice plants, 6 in total, were trimmed such that only the main stem remained in each pot. Then, the cleaned 6 potted rice plants were placed within a cage (30 × 30 × 60 cm^3^, made of iron wire frame and covered with 80-mesh nylon netting) and randomly arranged in a circular shape.

Thirty newly emerged macropterous adults (<24 h, 15 females and 15 males) from the *S. furcifera* colony reared on N0 rice plants were released from a 9 cm Petri dish placed into the center of the cage. After 96 h from release, the number of adults on each of the 6 plants was recorded, and the feeding preferences of each nitrogen fertility level were expressed as a percent of the adults settling on a specific plant. The feeding preference was calculated as: Feeding preference = The number of adults settling on a specific fertilizer level plant/Total number of tested adults × 100. This experiment was repeated 5 times and for each repetition, the location of the plants in the cage was rotated to avoid the effect of other factors.

The same arrangement was used to observe the adult’s oviposition preferences. Fifteen pairs of newly emerged macropterous adults from the *S. furcifera* colony reared on N0 rice plants were released for the oviposition preferences test. The experiment was replicated 5 times. After that, the number of first instars emerging and unhatched egg on a specific plant to count the total number of eggs laid. The leaf midribs and leaf sheaths of each nitrogen level rice were dissected under a binocular microscope to count the unhatched egg. The tests were performed in a climate chamber (RZH-260A, Hangzhou Huier Instrument Equipment Co., Ltd., Hangzhou, China) at 26 ± 1 °C, 70 ± 5% relative humidity (RH), and a photoperiod of 14L:10D. The oviposition preference was calculated as: Oviposition preference = The number of eggs laid on a specific fertilizer level plant/Total number of eggs laid × 100.

### 4.5. Life Parameters

A 4 cm-long rice leaf section cut from the top third or fourth leaf, starting 5 cm away from the leaf tip of a 35 DAT potted rice plant receiving a fertility treatment, was placed in a glass tube (1.0 cm in diameter, 7.5 cm in height) filled with 1 mL water. Then, one first instar nymph (<24 h) reared from the N0 rice plants was transferred into the glass tube and the opening was sealed with a sponge to prevent escape. The leaf section in the glass tube was replaced daily with a freshly cut leaf section from the same fertility treatment. Using the same procedure, rice leaves from all 6 fertility levels were prepared to evaluate the host’s impact on the life parameters of *S. furcifera*. All glass tubes containing rice leaf sections and individual nymphs were kept in a climate chamber at 26 ± 1 °C, 70 ± 5% RH, and a photoperiod of 14:10 (L:D) h. The nymphs’ developmental stage and mortality were recorded daily until they died or became adults, and the emerged adults were then sexed. The observation of nymphal survival was repeated at least 50 times for each of the 6 fertility levels.

The effects of nitrogen fertility on the longevity of newly emerged adults (<24 h) reared from the N0 rice plants were evaluated using the same arena and method as those for the nymphs, except that the newly emerged adults were transferred into the glass tubes at the beginning of the experiment. The longevity observation was replicated at least 35 times for each of the 6 fertility levels, and the ratio of males to females tended to be 1:1.

Newly emerged adult males and females (<24 h) reared from the rice plants of a fertility level were paired, and they were transferred into a glass tube (5 cm in diameter, 40 cm in height) housing a single 35 DAT rice plant of fertility treatment. The top of the glass tube was sealed with a sponge. The rice plant was replaced daily with a new plant from the same fertility treatment. Similar setups were prepared for all 6 fertility treatments. These experimental setups were used to observe the pre-oviposition and egg development periods. The observation of pre-oviposition was replicated four times for each treatment, with one pair of adults as a replicate. The egg developmental period observation was replicated at least 48 times, for each fertility treatment, with 1 egg as a replicate. The tests were performed in a climate chamber at 26 ± 1 °C, 70 ± 5% relative humidity (RH), and a photoperiod of 12L:12D.

### 4.6. Rice Physical Parameters

The above-ground heights of 35 DAT rice plants were measured, using a ruler, for 10 rice plants from each fertility level. To obtain the blade thickness and leaf areas, the following procedures were performed. From the main stem of a 35 DAT potted rice plant, the third leaf from the top was chosen. A 5 cm-long rice leaf section from the leaf tip was cut and discarded. Another 5 cm-long rice leaf section was cut from the same leaf and then weighed and photographed individually. The images were analyzed using the Image-Pro Plus v6.0 software to obtain the areas of the leaf blade sections. The leaf blade thickness was expressed as the weight per unit area (mg/cm^2^) according to Luo et al. [46]. The leaf blade width was calculated as Blade width = Blade area/Blade length. Four 5 cm leaf blade sections from the rice plants of each fertility treatment were used to obtain the blade area, thickness, and width data. All tests were replicated 10 times for each fertility treatment.

### 4.7. Rice Physiological Parameters

The chlorophyll content was positively correlated with nitrogen content and can be regarded as an indicator of the nitrogen content of plants [33]. The rice leaf tissue chlorophyll content was assessed using an electronic portable chlorophyll meter (SPAD-502, Minolta camera Co., Osaka, Japan) and expressed as the SPAD value. The mean of three SPAD readings measured by the chlorophyll meter was obtained from each of the top third leaves of ten 35 DAT rice plants of various fertility levels from 9:00 to 10:00 a.m. The tests were performed in a glasshouse, under natural light and temperature conditions, and repeated 10 times for each fertility treatment.

To obtain water content, the main stems from ten 35 DAT rice plants for each fertility level were cut from the base flush with the soil and weighed individually. These stems were immediately placed in an oven at 110 °C for 30 min and then at 80 °C for 48 h (when constant weight was achieved). The tests were repeated 5 times in each fertility treatment. The water content was calculated as: Water content = (Fresh weight − Dry weight)/Fresh weight × 100. The dry weight was regarded as dry matter mass.

The concentration of the soluble protein of 35 DAT rice plant leaves of various fertility levels was determined using the Coomassie Blue method [57], with bovine serum albumin as a standard, and the absorbance of the samples was measured at 595 nm using the UV-VIS Spectrophotometer. This procedure was repeated 5 times in each fertility treatment.

### 4.8. Statistical Analysis

The adult mortality ratio (Adult mortality ratio = Larval mortality/Total number of tested larvae × 100) in all the rice of the six nitrogen levels (N0, N50, N150, N200, N250, and N350) tested was transformed to probability units and analyzed using the logistic regression (Y = A/(1 + Exp (b − k X))), where X is the feeding days in each rice of different nitrogen levels and Y is the adult mortality ratio in probability units. The feeding days and adult mortality ratio data were fitted using probability analysis to estimate the number of feeding days that induced death in 50% and 90% of adults, respectively.

Percentage data (including feeding and oviposition preference, water content) were arcsine square-root-transformed, and all data fit normal distributions. Then, the homogeneity of variance of all data was tested before one-way analysis of variance (ANOVA). All data were subjected to ANOVA, followed by the least significant difference (LSD) multiple range test (*p* < 0.05) for determining the significant differences between the treatments. We investigated the relationships between *S. furcifera* and rice responses to nitrogen fertilizer application, and the relationships were then tested using the Pearson correlation coefficient. All the statistical analyses were performed using SPSS 17.0.

## 5. Conclusions

Nitrogen fertilizer can trigger bottom-up effects on rice and *S. furcifera*, favoring the colonization, survival, and development of this insect. Nitrogen inputs to rice increase the preference of infestation behaviors (feeding and oviposition), extend infestation time (adult lifespan), and shorten generation reproduction time (nymph, pre-oviposition, and egg period), causing a high infestation rate of *S. furcifera* populations in the paddies with high-nitrogen fertilizer. Various nitrogen inputs to rice plants affected the biological traits of the *S. furcifera* and these effects were due to the changes of rice physical and/or physiological indexes. Our results shed insight into how excess nitrogen fertilization shapes rice–planthopper nutritional interactions. Negative bottom-up effects in insufficient nitrogen input may help to optimize the IPM program of *S. furcifera* by nitrogen fertilizer manipulation. Further work will investigate on how nitrogen fertilization inputs shape rice–planthopper–natural enemies (i.e., *Cyrtorhinus lividipennis* and *Anagrus nilaparvatae*) nutritional interactions and further optimize agricultural practices by bottom-up effects.

## Figures and Tables

**Table 1:** Life parameters of *S. furcifera* feeding on rice with different nitrogen fertilizer applications.

Fertility Levels Rice	Nymph Period (days)	Adult Longevity (days)	Female Preoviposition Period (days)	Egg Period (days)	Total Pre-Oviposition Period (days)
N0	16.32 ± 0.31 a	9.19 ± 0.97 c	4.50 ± 0.50 a	7.46 ± 0.08 a	28.27 ± 0.5 a
N50	14.74 ± 0.27 b	11.46 ± 1.41 bc	4.00 ± 0.00 ab	7.40 ± 0.10 a	26.15 ± 0.2 b
N150	13.25 ± 0.19 d	14.96 ± 0.93 a	3.75 ± 0.25 abc	7.23 ± 0.05 b	24.23 ± 0.25 c
N200	13.98 ± 0.23 c	15.14 ± 0.84 a	2.75 ± 0.25 c	6.94 ± 0.05 c	23.67 ± 0.25 c
N250	13.30 ± 0.20 d	14.43 ± 1.07 a	3.25 ± 0.25 bc	6.72 ± 0.02 d	23.27 ± 0.25 c
N350	13.28 ± 0.24 d	13.26 ± 1.09 ab	3.75 ± 0.75 abc	6.81 ± 0.03 c	23.84 ± 0.75 c

Values are means ± standard error (SE). Different letters following the means in the same column denote significant difference at *p* < 0.05 via LSD multiple range tests. N0, N50, N150, N200, N250, and N350 represent the rice plants grown from the six fertility levels (0, 50, 150, 200, 250, and 350 kg/ha), respectively. Total pre-oviposition period is the duration from egg to first oviposition.

**Table 2:** Curve equation from a regression between adult cumulative mortality and feeding days on rice with different nitrogen fertilizer applications.

Fertility Levels Rice	Regression Equation	*R*	*R*_0.05_	*R*_0.01_
N0	Y = 96.719/(1 + Exp(2.834 − 0.369X))	0.994	0.355	0.456
N50	Y = 100.548/(1 + Exp(1.771 − 0.174X))	0.988	0.344	0.442
N150	Y = 99.076/(1 + Exp(3.737 − 0.266X))	0.996	0.339	0.436
N200	Y = 95.850/(1 + Exp(4.761 − 0.344X))	0.997	0.374	0.479
N250	Y = 99.038/(1 + Exp(3.148 − 0.233X))	0.998	0.349	0.449
N350	Y = 99.755/(1 + Exp(3.391 − 0.269X))	0.998	0.361	0.463

The regression equation was analyzed using SPSS. Y is the adult cumulative mortality of *S. furcifera*. X is the feeding days of *S. furcifera*. *R* is the coefficient of determination. *R*_0.05_ is the coefficient of determination at 95%, *R*_0.01_ is the coefficient of determination at 99%. *R* greater than *R*_0.05_ and *R*_0.01_ shows a strong relationship between adult cumulative mortality and feeding days. N0, N50, N150, N200, N250, and N350 represent the rice plants grown from the six fertility levels (0, 50, 150, 200, 250, and 350 kg/ha), respectively.

**Table 3:** Estimated parameters, confidence intervals, and *R*^2^ for a logistic model of adult cumulative mortality fitted to experimental data of feeding *S. furcifera* on rice with different nitrogen fertilizer applications.

Parameter	Cumulative Mortality Ratio		
	Estimated	(95% CI)	*R* ^2^
N0			
A	96.719	(94.734, 98.705)	0.988
b	2.834	(2.500, 3.168)	
k	0.369	(0.326, 0.413)	
N50			
A	100.548	(96.161, 104.936)	0.977
b	1.771	(1.546, 1.996)	
k	0.174	(0.149, 0.200)	
N150			
A	99.076	(96.573, 101.580)	0.992
b	3.737	(3.405, 4.069)	
k	0.266	(0.241, 0.292)	
N200			
A	95.850	(93.189, 98.511)	0.994
b	4.761	(4.319, 5.203)	
k	0.344	(0.310, 0.379)	
N250			
A	99.038	(97.055, 101.021)	0.996
b	3.148	(2.971, 3.324)	
k	0.233	(0.218, 0.248)	
N350			
A	99.755	(97.642, 101.867)	0.996
b	3.391	(3.168, 3.614)	
k	0.269	(0.250, 0.289)	

A, b, and k represent the parameters in the logistic equation (Y = A/(1 + Exp (b − k X))). 95% CI, 95% confidence intervals. *R*^2^, the coefficient of determination. N0, N50, N150, N200, N250, and N350 represent the rice plants grown from the six fertility levels (0, 50, 150, 200, 250, and 350 kg/ha), respectively.

**Table 4:** Time estimates for 50% and 90% of adult cumulative mortality.

Fertility Levels Rice	Time Estimates			
	50% Adult Cumulative Mortality		90% Adult Cumulative Mortality	
	Days	(95% CI)	Days	(95% CI)
N0	7.9	(5.1, 10.6)	14.7	(9.3, 20.1)
N50	10.1	(7.1, 13.2)	22.5	(16.5, 28.5)
N150	14.1	(12.0, 16.3)	22.7	(18.5, 26.9)
N200	14.1	(11.7, 16.5)	21.8	(17.1, 26.5)
N250	13.6	(12.1, 15.1)	23.4	(20.4, 26.3)
N350	12.6	(10.8, 14.5)	20.9	(17.2, 24.6)

CI, confidence interval. N0, N50, N150, N200, N250, and N350 represent the rice plants grown from the six fertility levels (0, 50, 150, 200, 250, and 350 kg/ha), respectively.

**Table 5:** Responses of rice physical parameters to nitrogen fertilizer application.

Fertility Levels Rice	Plant Height (cm)	Leaf Area (cm^2^)	Leaf Width (cm)	Leaf Thickness (mg/cm^2^)
N0	34.3 ± 2.0 b	2.0 ± 0.2 d	0.4 ± 0.0 d	12.2 ± 0.3 a
N50	39.1 ± 1.9 b	2.2 ± 0.1 cd	0.4 ± 0.0 cd	11.0 ± 0.7 b
N150	49.2 ± 2.7 a	2.8 ± 0.1 ab	0.6 ± 0.0 ab	10.1 ± 0.3 bc
N200	48.9 ± 2.2 a	2.4 ± 0.1 bc	0.5 ± 0.0 bc	10.6 ± 0.5 bc
N250	49.9 ± 1.9 a	2.9 ± 0.1 a	0.6 ± 0.0 a	9.7 ± 0.2 c
N350	46.8 ± 2.5 a	3.1 ± 0.1 a	0.6 ± 0.0 a	10.0 ± 0.2 bc

Values are means ± standard error (SE). Different letters following the means in the same column denote significant difference at *p* < 0.05 via LSD multiple range tests. N0, N50, N150, N200, N250, and N350 represent the rice plants grown from the six fertility levels (0, 50, 150, 200, 250, and 350 kg/ha), respectively.

**Table 6:** Responses of rice physiological parameters to nitrogen fertilizer application.

Fertility Levels Rice	Chlorophyll Content (SPAD)	Water Content (%)	Dry Matter Mass (mg)	Soluble Protein Content (mg/g)
N0	25.8 ± 0.5 e	77.0 ± 0.4 c	311.9 ± 29.1 d	14.2 ± 0.5 d
N50	28.7 ± 0.7 d	78.9 ± 0.8 b	1077.5 ± 108.6 c	15.6 ± 0.1 c
N150	33.5 ± 0.7 c	80.3 ± 0.5 ab	1615.9 ± 84.5 ab	15.5 ± 0.4 c
N200	34.9 ± 0.8 bc	80.8 ± 0.4 a	1840.5 ± 87.9 a	16.3 ± 0.3 bc
N250	35.9 ± 0.9 ab	81.7 ± 0.4 a	1305.7 ± 214.0 bc	16.7 ± 0.4 ab
N350	37.5 ± 0.8 a	80.8 ± 0.7 a	1707.8 ± 122.8 a	17.7 ± 0.1 a

Values are means ± standard error (SE). SPAD represents rice plants’ chlorophyll content. Different letters following the means in the same column denote significant difference at *p* < 0.05 via LSD multiple range tests. Percentage data were arcsine square-root-transformed, and homogeneity of variance of all data was tested before ANOVA. N0, N50, N150, N200, N250, and N350 represent the rice plants grown from the six fertility levels (0, 50, 150, 200, 250, and 350 kg/ha), respectively.

**Table 7:** Pearson’s correlation coefficients and *p*-values between *S. furcifera* and rice responses to different nitrogen fertilizer applications.

*S. furcifera* Responses Parameters	Rice Responses Parameters							
	Physical Parameters				Physiological Parameters			
	Plant Height	Leaf Area	Leaf Width	Leaf Thickness	Chlorophyll Content (SPAD)	Water Content	Dry Matter Mass	Soluble Protein Content
Feeding selection ratio	0.716 (0.110)	0.512 (0.299)	0.512 (0.299)	−0.574 (0.234)	0.817 (0.047)	0.771 (0.073)	0.763 (0.078)	0.794 (0.059)
Oviposition selection ratio	0.539 (0.270)	0.823 (0.044)	0.823 (0.044)	−0.73 (0.100)	0.794 (0.059)	0.712 (0.113)	0.470 (0.347)	0.898 (0.015)
Nymph period	−0.940 (0.005)	−0.920 (0.009)	−0.920 (0.009)	−0.984 (0.000)	−0.923 (0.009)	−0.940 (0.005)	−0.881 (0.020)	−0.826 (0.043)
Adult longevity	0.979 (0.001)	0.687 (0.131)	0.687 (0.131)	−0.845 (0.034)	0.841 (0.036)	0.914 (0.011)	0.910 (0.012)	0.660 (0.154)
Female preoviposition period	−0.818 (0.047)	−0.413 (0.416)	−0.413 (0.416)	0.618 (0.191)	−0.728 (0.101)	−0.823 (0.044)	−0.784 (0.065)	−0.638 (0.173)
Egg period	−0.802 (0.055)	−0.804 (0.054)	−0.804 (0.054)	0.804 (0.054)	−0.924 (0.008)	−0.896 (0.016)	−0.668 (0.147)	−0.886 (0.019)

The correlations were then tested using Pearson’s correlation coefficient, *p*-values shown in parentheses. The correlation level for significance is set to *p* < 0.05.

## References

[1] Han P., Desneux N., Michel T., Le Bot J., Seassau A., Wajnberg E., Amiens-Desneux E., Lavoir A.V. (2016). Does plant cultivar difference modify the bottom-up effects of resource limitation on plant-insect herbivore interactions?. J. Chem. Ecol..

[2] Randlkofer B., Obermaier E., Hilker M., Meiners T. (2010). Vegetation complexity-The influence of plant species diversity and plant structures on plant chemical complexity and arthropods. Basic Appl. Ecol..

[3] Ballhorn D.J., Kautz S., Jensen M., Schmitt I., Heil M., Hegeman A.D. (2011). Genetic and environmental interactions determine plant defences against herbivores. J. Ecol..

[4] Chen Y.G., Olson D.M., Ruberson J.R. (2010). Effects of nitrogen fertilization on tritrophic interactions. Arthropod Plant Interact..

[5] Han P., Lavoir A.V., Le Bot J., Amiens-Desneux E., Desneux N. (2014). Nitrogen and water availability to tomato plants triggers bottom-up effects on the leafminer *Tuta absoluta*. Sci. Rep..

[6] Han P., Bearez P., Adamowicz S., Lavoir A.V., Amiens-Desneux E., Desneux N. (2015). Nitrogen and water limitations in tomato plants trigger negative bottom-up effects on the omnivorous predator Macrolophus pygmaeus. J. Pest. Sci..

[7] Han P., Becker C., Sentis A., Rostas M., Desneux N., Lavoir A.V. (2019). Global change-driven modulation of bottom-up forces and cascading effects on biocontrol services. Curr. Opin. Insect. Sci..

[8] Blazheyski S., Kalaitzaki A.P., Tsagkarakis A.E. (2018). Impact of nitrogen and potassium fertilization regimes on the biology of the tomato leaf miner Tuta absoluta. Entomol Gen..

[9] Ye Z.P., Vollhardt I.M.G., Parth N., Rubbmark O., Traugott M. (2018). Facultative bacterial endosymbionts shape parasitoid food webs in natural host populations: A correlative analysis. J. Anim. Ecol..

[10] William J., Mattson J. (1980). Herbivory in relation to plant nitrogen content. Annu. Rev. Ecol. Evol. Syst..

[11] Schetter T.A., Lochmiller R.L., Leslie D.M., Engle D.M., Payton M.E. (1998). Examination of the nitrogen limitation hypothesis in non-cyclic populations of cotton rats (Sigmodon hispidus). J. Anim. Ecol..

[12] Kurze S., Heinken T., Fartmann T. (2018). Nitrogen enrichment in host plants increases the mortality of common Lepidoptera species. Oecologia.

[13] Veromann E., Toome M., Kanaste A., Kaasik R., Copolovici L., Flink J., Kovacs G., Narits L., Luik A., Niinemets U. (2013). Effects of nitrogen fertilization on insect pests, their parasitoids, plant diseases and volatile organic compounds in Brassica napus. Crop Prot..

[14] Huang J.R., Sun J.Y., Liao H.J., Liu X.D. (2015). Detection of brown planthopper infestation based on SPAD and spectral data from rice under different rates of nitrogen fertilizer. Precis. Agric..

[15] Jayasimha G.T., Nalini R., Chinniah C., Muthamilan M., Mini M.L. (2015). Evaluation of biochemical constituents in healthy and brown planthopper, *Nilaparvata lugens* (Stal.) (Hemiptera: Delphacidae) damaged rice plants. Curr. Biot..

[16] Wang L.Y., Gao F., Reddy G.V.P., Zhao Z.H. (2020). Optimization of nitrogen fertilizer application enhances biocontrol function and net income. J. Econ. Entomol..

[17] Coqueret V., Le Bot J., Larbat R., Desneux N., Robin C., Adamowicz S. (2017). Nitrogen nutrition of tomato plant alters leafminer dietary intake dynamics. J. Insect. Physiol..

[18] Aqueel M.A., Raza A.B.M., Balal R.M., Shahid M.A., Mustafa I., Javaid M.M., Leather S.R. (2015). Tritrophic interactions between parasitoids and cereal aphids are mediated by nitrogen fertilizer. Insect Sci..

[19] Kyto M., Niemela P., Larsson S. (1996). Insects on trees: Population and individual response to fertilization. Oikos.

[20] Fischer K., Fiedler K. (2000). Response of the copper butterfly *Lycaena tityrus* to increased leaf nitrogen in natural food plants: Evidence against the nitrogen limitation hypothesis. Oecologia.

[21] Cease A.J., Elser J.J., Ford C.F., Hao S.G., Kang L., Harrison J.F. (2012). Heavy livestock grazing promotes locust outbreaks by lowering plant nitrogen content. Science.

[22] Le Gall M., Word M.L., Thompson N., Beye A., Cease A.J. (2020). Nitrogen fertilizer decreases survival and reproduction of female locusts by increasing plant protein to carbohydrate ratio. J. Anim. Ecol..

[23] Amirjani M.R. (2011). Effect of salinity stress on growth, sugar content, pigments and enzyme activity of rice. Int. J. Bot..

[24] Zhu Z.L., Chen D.L. (2002). Nitrogen fertilizer use in China—Contributions to food production, impacts on the environment and best management strategies. Nutr. Cycl. Agroecosyst..

[25] Zhang W.F., Ma L., Huang G.Q., Wu L., Chen X.P., Zhang F.S. (2013). The development and contribution of nitrogenous fertilizer in China and challenges faced by the country. Sci. Agric. Sin..

[26] Ju X.T., Gu B.J. (2014). Status-quo, problem and trend of nitrogen fertilization in China. J. Plant Nutr. Fert..

[27] Yang L., Han Y.Q., Li P., Wen L.Z., Hou M.L. (2017). Silicon amendment to rice plants impairs sucking behaviors and population growth in the phloem feeder *Nilaparvata lugens* (Hemiptera: Delphacidae). Sci. Rep..

[28] Zhou X.J., Xu H.X., Zheng X.S., Yang Y.J., Chen L.W., He J.H., Lu Z.X. (2012). Population dynamics of white-backed planthopper and its predatory spiders in hybrid rice fields with different nitrogen regimes. Acta Agric. Zhejiangensis.

[29] Zmurchok C., de Vries G. (2018). Direction-dependent interaction rules enrich pattern formation in an individual-based model of collective behavior. PLoS ONE.

[30] Thompson J.N. (1988). Evolutionary ecology of the relationship between oviposition preference and performance of offspring in phytophagous insects. Entomol. Exp. Appl..

[31] Stein S.J., Price P.W. (1995). Relative Effects of plant-resistance and natural enemies by plant developmental age on sawfly (Hymenoptera, Tenthredinidae) preference and performance. Environ. Entomol..

[32] Kagata H., Ohgushi T. (2001). Preference and performance linkage of a leaf-mining moth on different Salicaceae species. Popul. Ecol..

[33] Netto A.T., Campostrini E., de Oliveira J.G., Bressan-Smith R.E. (2005). Photosynthetic pigments, nitrogen, chlorophyll a fluorescence and SPAD-502 readings in coffee leaves. Sci. Hortic Amst..

[34] Han P., Niu C.Y., Lei C.L., Cui J.J., Desneux N. (2010). Use of an innovative T-tube maze assay and the proboscis extension response assay to assess sublethal effects of GM products and pesticides on learning capacity of the honey bee *Apis mellifera* L.. Ecotoxicology.

[35] Islam M.N., Hasanuzzaman A.M., Zhang Z.F., Zhang Y., Liu T.X. (2017). High level of nitrogen makes tomato plants releasing less volatiles and attracting more *Bemisia tabaci* (Hemiptera: Aleyrodidae). Front. Plant Sci..

[36] Fallahpour F., Ghorbani R., Mahallati M.N., Hosseini M. (2015). Demographic parameters of *Lipaphis erysimi* on canola cultivars under different nitrogen fertilization regimes. J. Agric. Sci. Technol..

[37] Golizadeh A., Kamali K., Fathipour Y., Abbasipour H. (2009). Life table of the diamondback moth, *Plutella xylostella* (L.) (Lepidoptera: Plutellidae) on five cultivated brassicaceous host plants. J. Agric. Sci. Technol..

[38] Daugherty M.P. (2011). Host plant quality, spatial heterogeneity, and the stability of mite predator-prey dynamics. Exp. Appl. Acarol..

[39] Facknath S., Lalljee B. (2005). Effect of soil-applied complex fertiliser on an insect-host plant relationship: *Liriomyza trifolii* on Solanum tuberosum. Entomol. Exp. Appl..

[40] Yang N.B., Wu S.R., Shen L.B., Zhang S.Z., Yang B.P. (2014). A review on plant resistance to insect pests. Chin. J. Trop. Agric..

[41] Hosseini M., Ashouri A., Enkegaard A., Goldansaz S.H., Nassiri Mahalati M., Hosseininaveh V. (2010). Performance and population growth rate of the cotton aphid, and associated yield losses in cucumber, under different nitrogen fertilization regimes. Int. J. Pest Manag..

[42] Hosseini R.S., Madadi H., Hosseini M., Delshad M., Dashti F. (2015). Nitrogen in Hydroponic Growing Medium of Tomato Affects the Demographic Parameters of *Trialeurodes vaporariorum* (Westwood) (Hemiptera: Aleyrodidae). Neotrop. Entomol..

[43] Zanotto F.P., Simpson S.J., Raubenheimer D. (1993). The regulation of growth by locusts through post-ingestive compensation for variation in the levels of dietary protein and carbohydrate. Physiol. Entomol..

[44] Han Y.Q., Lei W.B., Wen L.Z., Hou M.L. (2015). Silicon-mediated resistance in a susceptible rice variety to the rice leaf folder, *Cnaphalocrocis medinalis* Guenee (Lepidoptera: Pyralidae). PLoS ONE.

[45] Zheng W.J., Liu Z.H., Zhang Y.Z., Liu X., Wang C.H., Zhao J.M. (2009). Effects of the major nutritional substances and micro-structure of rice plants on the host preference of the small brown planthopper, *Laodelphax striatellus*. Acta Phytophyl. Sin..

[46] Luo J.Y., Cui J.J., Huang Q. (2011). The relationship between the content of cotton leaf chlorophyll and waxiness and leaf thickness and the cotton resistance to *Apolygus lucorum*. Acta Phytophyl. Sin..

[47] Butter N.S., Vir B.K. (1989). Morphological Basis of Resistance in Cotton to the White fly *Bemisia Tabaci*. Phytoparasitica.

[48] Lin F.M., Wu D., Lu Y.H., Zhang Y.J., Wang M., Wu K.M., Guo Y.Y. (2010). Effects of leaf thickness and gossypol gland density of cotton on its resistance to *Apolygus lucorum* (Hemiptera Miridae). Acta Entomol. Sin..

[49] Jiang S., Liu T., Yu F., Li T., Parajulee M.N., Zhang L., Chen F. (2016). Feeding behavioral response of cotton aphid, Aphis gossypii, to elevated CO2: EPG test with leaf microstructure and leaf chemistry. Entomol. Exp. Appl..

[50] Walker G.P. (1985). Stylet penetration by the bayberry whitefly, as affected by leaf age in lemon, Citrus limon. Entomol. Exp. Appl..

[51] Huang C.W., Feng B.S. (1993). Quantitative aspects of feeding activity in the white-back planthopper *Sogatella furcifera* and the brown planthopper. Acta Entomol. Sinica.

[52] Qin H.G., Ye Z.X., Huang R.H. (1991). Study on effect of fertilization on the field population density of *Sogatella furcifera* and the rice yield. Acta Agric. Univ. Jiangxiensis.

[53] Price P. (1991). The plant vigor hypothesis and herbivore attack. Oikos.

[54] White T.C.R. (1993). The Inadequate Environment: Nitrogen and the Abundance of Animals.

[55] Dong Y.C., Han P., Niu C.Y., Zappala L., Amiens-Desneux E., Bearez P., Lavoir A.V., Biondi A., Desneux N. (2018). Nitrogen and water inputs to tomato plant do not trigger bottom-up effects on a leafminer parasitoid through host and non-host exposures. Pest Manag. Sci..

[56] Che S.G., Zhao B.Q., Li Y.T., Yuan L., Li W., Lin Z.A., Hu S.W., Shen B. (2015). Review grain yield and nitrogen use efficiency in rice production regions in China. J. Integr. Agric..

[57] Bradford M.M. (1976). A rapid and sensitive method for the quantitation of microgram quantities of protein utilizing the principle of protein-dye binding. Anal. Biochem..

